# Supplementary material for: Nano-imaging trace elements at organelle levels in substantia nigra overexpressing α-synuclein to model Parkinson’s disease
Source: Commun Biol. 2020 Jul 9;3:364. doi: 10.1038/s42003-020-1084-0 (PMC7347932; doi:10.1038/s42003-020-1084-0)
Supplement: Supplementary file 1 — Supplementary Information [file 42003_2020_1084_MOESM1_ESM.pdf]

SUPPLEMENTARY INFORMATION

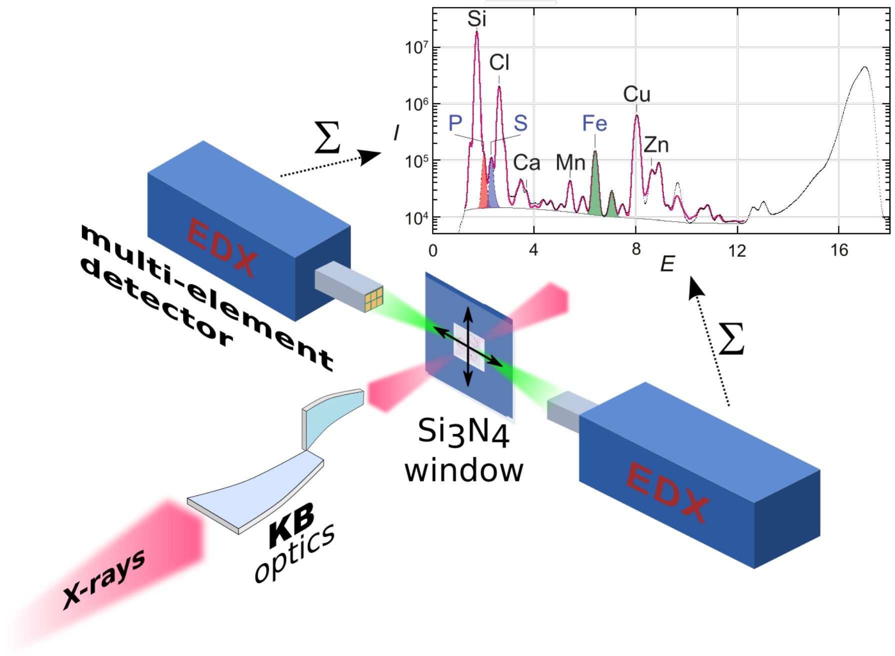

Supplementary Figure 1 – Schematic of the ID16A XRF set-up. X-rays produced by a single line undulator (not shown) are focused by multilayer coated fixed curvature Kirkpatrick-Baez optics to 23 × 37 nm^2 focus size. The sample is scanned through the focus at normal incidence while the X-ray fluorescence signal is collected by one or two 6-element energy dispersive detectors. The thin tissue section is mounted on a silicon nitride window of 500 nm membrane thickness. As typical spectrum (summed over all detector elements and pixels) is shown at the top right, together with the fit and the identification of the most important elements.

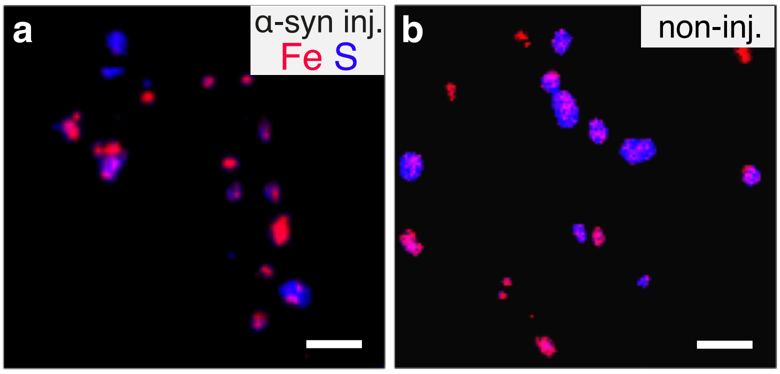

Supplementary Figure 2 – Analysis of iron and sulfur-rich granules within the cytoplasm. (**a, b**) Same nano-XRF stacks of fig. 5 a, b showing the Fe and S mass fractions but differently color-coded red/blue in the neuronal cytoplasm for the AAV-α-syn injected (**a**) and control non-injected (**b**) conditions. Scale bars: 1 µm.
