# Supplementary material for: Nano-imaging trace elements at organelle levels in substantia nigra overexpressing α-synuclein to model Parkinson’s disease
Source: Commun Biol. 2020 Jul 9;3:364. doi: 10.1038/s42003-020-1084-0 (PMC7347932; doi:10.1038/s42003-020-1084-0)
Supplement: Supplementary file 3 — Reporting Summary [file 42003_2020_1084_MOESM3_ESM.pdf]

nature research

Corresponding author(s): Bernard Schneider, Alexandre Simionovici

Last updated by author(s): Jun 17, 2020

Reporting Summary

Nature Research wishes to improve the reproducibility of the work that we publish. This form provides structure for consistency and transparency in reporting. For further information on Nature Research policies, see our Editorial Policies and the Editorial Policy Checklist.

Statistics

For all statistical analyses, confirm that the following items are present in the figure legend, table legend, main text, or Methods section.

n/a	Confirmed	
☐	☒	The exact sample size (n) for each experimental group/condition, given as a discrete number and unit of measurement
☐	☒	A statement on whether measurements were taken from distinct samples or whether the same sample was measured repeatedly
☒	☐	The statistical test(s) used AND whether they are one- or two-sided<br>*Only common tests should be described solely by name; describe more complex techniques in the Methods section.*
☒	☐	A description of all covariates tested
☒	☐	A description of any assumptions or corrections, such as tests of normality and adjustment for multiple comparisons
☐	☒	A full description of the statistical parameters including central tendency (e.g. means) or other basic estimates (e.g. regression coefficient) AND variation (e.g. standard deviation) or associated estimates of uncertainty (e.g. confidence intervals)
☒	☐	For null hypothesis testing, the test statistic (e.g. F, t, r) with confidence intervals, effect sizes, degrees of freedom and P value noted<br>*Give P values as exact values whenever suitable.*
☒	☐	For Bayesian analysis, information on the choice of priors and Markov chain Monte Carlo settings
☒	☐	For hierarchical and complex designs, identification of the appropriate level for tests and full reporting of outcomes
☒	☐	Estimates of effect sizes (e.g. Cohen's d, Pearson's r), indicating how they were calculated

Our web collection on statistics for biologists contains articles on many of the points above.

Software and code

Policy information about availability of computer code

Data collection	ESRF proprietary acquisition software based on the SPEC CSS package. SPEC is a UNIX-based software package for instrument control and data acquisition widely used at synchrotrons and labs around the world. CSS stands for Certified Scientific Software, USA.
Data analysis	PyMCA and ImageJ. ImageJ is a Java-based image processing program developed at the National Institutes of Health and the Laboratory for Optical and Computational Instrumentation.

For manuscripts utilizing custom algorithms or software that are central to the research but not yet described in published literature, software must be made available to editors and reviewers. We strongly encourage code deposition in a community repository (e.g. GitHub). See the Nature Research guidelines for submitting code & software for further information.

Data

Policy information about availability of data

All manuscripts must include a data availability statement. This statement should provide the following information, where applicable:

- Accession codes, unique identifiers, or web links for publicly available datasets
- A list of figures that have associated raw data
- A description of any restrictions on data availability

Raw data were generated at the ESRF European synchrotron large-scale facility. Derived data supporting the findings of this study are available upon request to the corresponding authors.

Field-specific reporting

Please select the one below that is the best fit for your research. If you are not sure, read the appropriate sections before making your selection.

☒ Life sciences ☐ Behavioural & social sciences ☐ Ecological, evolutionary & environmental sciences

For a reference copy of the document with all sections, see nature.com/documents/nr-reporting-summary-flat.pdf

Life sciences study design

All studies must disclose on these points even when the disclosure is negative.

Sample size	Sample size was maximized according to the available beamtime, which is the limiting factor for synchrotron experiments.
Data exclusions	There was no data exclusion in this study.
Replication	Data were replicated across three imaging sessions using samples derived from two animals per condition. For quantification of the elemental composition, the replicates were derived from the three imaging sessions.
Randomization	N/A
Blinding	N/A

Reporting for specific materials, systems and methods

We require information from authors about some types of materials, experimental systems and methods used in many studies. Here, indicate whether each material, system or method listed is relevant to your study. If you are not sure if a list item applies to your research, read the appropriate section before selecting a response.

Materials & experimental systems

n/a	Involved in the study	
☒	☐	Antibodies
☒	☐	Eukaryotic cell lines
☒	☐	Palaeontology and archaeology
☐	☒	Animals and other organisms
☒	☐	Human research participants
☒	☐	Clinical data
☒	☐	Dual use research of concern

Methods

n/a	Involved in the study	
☒	☐	ChIP-seq
☒	☐	Flow cytometry
☒	☐	MRI-based neuroimaging

Animals and other organisms

Policy information about studies involving animals; ARRIVE guidelines recommended for reporting animal research

Laboratory animals	Three young adult female Sprague-Dawley rats weighing approximately 200 g
Wild animals	N/A
Field-collected samples	N/A
Ethics oversight	All procedures were approved by a local ethics committee and performed in accordance with the Swiss legislation and the European Community Council directive (86/609/EEC) regulating care and use of laboratory animals.

Note that full information on the approval of the study protocol must also be provided in the manuscript.
